# Effective Label-Free Sorting of Multipotent Mesenchymal Stem Cells from Clinical Bone Marrow Samples

**DOI:** 10.3390/bioengineering9020049

**Published:** 2022-01-22

**Authors:** Silvia Zia, Carola Cavallo, Ilaria Vigliotta, Valentina Parisi, Brunella Grigolo, Roberto Buda, Pasquale Marrazzo, Francesco Alviano, Laura Bonsi, Andrea Zattoni, Pierluigi Reschiglian, Barbara Roda

**Affiliations:** 1 Stem Sel Ltd., Viale Giuseppe Fanin 48, 40127 Bologna, Italy; silvia.zia@stemsel.it (S.Z.); or andrea.zattoni@unibo.it (A.Z.); or pierluigi.reschiglian@unibo.it (P.R.); 2 Laboratory RAMSES, Research & Innovation Technology Department, IRCCS Istituto Ortopedico Rizzoli, 40136 Bologna, Italy; carola.cavallo@ior.it (C.C.); valentina.parisi@ior.it (V.P.); brunella.grigolo@ior.it (B.G.); 3 Unit of Histology, Embryology and Applied Biology, Department of Experimental, Diagnostic and Specialty Medicine, University of Bologna, Via Belmeloro 8, 40126 Bologna, Italy; ilaria.vigliotta2@unibo.it (I.V.); pasquale.marrazzo2@unibo.it (P.M.); francesco.alviano@unibo.it (F.A.); laura.bonsi@unibo.it (L.B.); 4 1st Orthopaedic and Traumatologic Clinic, IRCCS Istituto Ortopedico Rizzoli, 40136 Bologna, Italy; roberto.buda@unich.it; 5 Department of Chemistry “G. Ciamician”, University of Bologna, Via Selmi 2, 40126 Bologna, Italy

**Keywords:** mesenchymal stem cells, bone marrow, label-free separation, cell selection, osteoarticular regeneration

## Abstract

Mesenchymal stem cells (MSC) make up less than 1% of the bone marrow (BM). Several methods are used for their isolation such as gradient separation or centrifugation, but these methodologies are not direct and, thus, plastic adherence outgrowth or magnetic/fluorescent-activated sorting is required. To overcome this limitation, we investigated the use of a new separative technology to isolate MSCs from BM; it label-free separates cells based solely on their physical characteristics, preserving their native physical properties, and allows real-time visualization of cells. BM obtained from patients operated for osteochondral defects was directly concentrated in the operatory room and then analyzed using the new technology. Based on cell live-imaging and the sample profile, it was possible to highlight three fractions (F1, F2, F3), and the collected cells were evaluated in terms of their morphology, phenotype, CFU-F, and differentiation potential. Multipotent MSCs were found in F1: higher CFU-F activity and differentiation potential towards mesenchymal lineages compared to the other fractions. In addition, the technology depletes dead cells, removing unwanted red blood cells and non-progenitor stromal cells from the biological sample. This new technology provides an effective method to separate MSCs from fresh BM, maintaining their native characteristics and avoiding cell manipulation. This allows selective cell identification with a potential impact on regenerative medicine approaches in the orthopedic field and clinical applications.

## 1. Introduction

Bone marrow (BM) is one of the most studied sources of MSCs, whose therapeutic potential has been explored in several diseases, proving its efficacy in clinical trials, from heart failure to grafts versus host disease [1] and other pathologies. In the orthopedic field, the use of bone marrow concentrate (BMC) in substitution of or addition to the procedure of marrow stimulation, provides interesting results because it contains not only hematopoietic stem cells (HSCs) and mesenchymal stem cells (MSCs) as a source for regenerating tissues but also accessory cells that support angiogenesis and vasculogenesis by producing several growth factors [2]. It is important to notice that BM clot as a 3D environment supports the chondrogenesis and osteogenesis of MSCs, making this system ideal for cartilage and/or bone repair. To understand its regenerative activity, studies are conducted to explore the biology and characteristics of each cell component. Once BM is harvested by aspiration, different types of cells are collected simultaneously and the isolation procedures can differ in the yield, but do not alter the balance of the different cell types [3]. Cells can be enriched by a density gradient system and centrifugal concentrator. Once mononuclear cells are concentrated, the gold-standard isolation technique is to exploit the plastic adherence properties of MSCs that lead to a heterogeneous population in culture, with plastic and non-plastic adherent cells, whose co-culture interactions and functional properties are unknown. The density gradient and antibody labelling system require several hours of labour-intensive multi-step processing, consisting of centrifugation and washing steps, potentially resulting in a significant amount of target stem cell loss [4]. Antibody-labelled techniques necessitate a high number of cells and the expression of multiple markers, which are not always available and can be co-expressed in different cell populations. The identity of the “best” marker for MSC identification, in both human and mouse systems, remains debated [5]. The International Society for Cellular Therapy has proposed a set of criteria to define MSCs [6], but the list of biomarkers is not fulfilling, and other tests, such as differentiation towards different lineages and immunomodulation, need to be taken into account to prove the stemness potential. The inability to isolate a pure population of MSCs has consequences in different fields, from basic research to therapeutic applications caused by differences in the proliferation rate, distinct differentiation program, and self-renewal capacity of each cell subset [7,8,9]. Therefore, new isolation approaches are needed in order to homogenize the cell culture among patients and centres.

In this work, we demonstrated the use of a new technique (Celector^®^, Stem Sel Ltd., Bologna, Italy) to isolate homogeneous stem cells directly from a clinical sample, the BMC, in a label-free mode. Celector^®^ is able to analyze cells based on differences in their physical properties, such as dimension, morphology, density, and membrane definition, to sort cells from in vitro culture to raw or tissue-digested sample. The characterization of MSCs isolated from different tissues was already demonstrated, together with the enrichment of homogeneous stem cell samples from an MSC heterogeneous cell population [10]. The label-free separation method was also successfully applied to raw clinical samples such as fetal membrane for the separation of MSC from epithelial cells obtained after tissue digestion [11], or to lipoaspirate for the isolation of MSC [12]. The soft/gentle fractionation mechanism guarantees the preservation of the cell native physical features, as there is no contact between the eluted cells and the separation device; therefore, viable, intact cells can be collected and used. The separation is achieved in a fluidic system provided of a micro-camera connected to the counting software, which generate the fractogram as output data, and allowed the visualization and image analysis of cells during the separation.

This study aimed to explore the dual features of this new technique applied to raw samples: analyze cells from BMC, which are known to have a broader size distribution and heterogeneous features, with the goal to identify and sort homogeneous and high quality MSCs; investigate quality control feature to forecast the presence of MSCs in samples obtained from patients operated for musculoskeletal pathologies.

A method based on Celector^®^ was developed and applied to BMC samples from patients. Selected cell fractions were collected and were tested in terms of colony-forming units, the expression of typical MSCs phenotype, and the differentiation toward chondrogenic and osteogenic sense, in order to envisage their specific clinical use.

## 2. Materials and Methods

### 2.1. Sample Harvesting and Preparation

BM was obtained from the iliac crest of 8 patients (mean age 35 [15 ÷ 66 y/o]; 3 females and 5 males) who underwent autologous cell transplantation for the treatment of osteo-chondral defects. The inclusion and exclusion criteria for the patients operated on with this treatment were the following. Inclusion criteria: osteochondral lesions grade III or IV of the International Cartilage Regeneration & Joint Preservation Society (ICRS) and lesion dimension > 1.5 cm^2^. Exclusion criteria: osteoarthritis, malalignments or ankle instability, infectious disease or presence of haematological or rheumatological diseases and coagulation disorders. The Ethical Committee of the Institution approved the human protocol for this study (number: 004350). All investigations were conducted in conformity with ethical principles of research, and written informed consent was signed by all the patients enrolled into the study. BM was harvested following standard procedure [13] (Supplementary Material) and concentrated using the kit IOR-G1 (Novagenit, Mezzolombardo, Italy) to reduce volume from 60 to 6 mL directly in the operating room, removing most of the red blood cells (RBC) and plasma. Mononuclear cells were counted using crystal violet solution (Sigma Aldrich, St. Louis, MO, USA) to exclude red blood cells and 3 × 10^6^ cells of BMC left unused from clinical treatment was processed using the gold-standard procedure by direct plating on tissue culture dish. Cells were plated at a cell density of 20,000 cells/cm^2^ and cultured in expansion medium made of minimum essential medium Eagle alpha modification (α-MEM, Gibco, Rockville, MD, USA) supplemented with 15% of foetal bovine serum (FBS, Euroclone S.p.A., Milan, Italy) and 1% penicillin-streptomycin (Gibco). Cells were grown in a 5% CO_2_ humidified chamber. Approximately 8 × 10^6^ of the BMC was employed for the separation using Celector^®^.

Two additional samples were used for the method development to investigate the best sample preparation for MSC-isolation protocol by Celector^®^. BM samples were equally divided into two parts; one part was processed using density gradient technique Ficoll^®^ following the manufacturer’s instructions (Ficoll^®^ Paque, Sigma Aldrich, Darmstadt, Germany), and the second one was concentrated using the kit IOR-G1.

### 2.2. Celector^®^ Instrument

**Figure 1:**
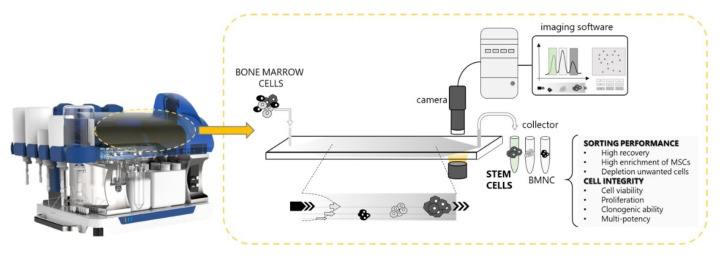
Schematic representation of the use of Celector^®^ in bone marrow processing. Bone marrow concentrate is injected into the inlet of the separation channel filled with mobile phase and eluted through it, acquiring different velocities related to their physical properties. A camera connected to the imaging software that plots the number of counted cells vs. time (fractogram) visualizes cells. Finally, cells are collected at the outlet and divided into different tubes according to the sample’s fractogram.

Celector^®^ instrument consists of a fluidic system and a biocompatible capillary separation device implementing a patented technology (IT1371772, US8263359 and CA2649234). The separation device is made of inert and biocompatible plastic material of 40 cm length, 4 cm width, and 250 µm thickness connected to the fluidic system. A micro-camera detector placed at the exit of the separation channel (USB 2.0 board-level camera —mvBlueFOX-MLC, Matrix Vision, Oppenweiler, Germany) monitors the elution process, generating a recorded plot of the eluted cell number as a function of time (fractogram) and for frames acquisition. A fraction collector is connected to the separation device. The instrument was placed inside a laminar flow cabinet to provide sterile working conditions. A schematic view of the instrumentation setup and fractionation procedure is reported in Figure 1.

### 2.3. Fractionation Principle and Procedure

The separation is obtained in a rectangular-shaped capillary device, 4 cm wide and 250 µm high, where cell suspensions are eluted through a laminar flow of mobile phase (Figure 1). Injected cells reach a specific position across the channel thickness during transportation due to the combined action of gravity, acting perpendicularly to the flow, and opposing the lift forces that depend on the morphological features of the sample. Cells at a specific position in the channel acquire well-defined velocities and, consequently, eluted at specific times. Cell suspension is injected into the system at a flow rate of 1 mL/min; subsequently, the flow is interrupted to allow sample relaxation, a process necessary to make analytes reach an equilibrium position along the channel thickness as a response of the external field. The relaxation time depends on the cell type, and it is usually a few minutes long for mammalian cells. Finally, sample elution was carried out by reactivating the flow of mobile phase [14,15].

The fractionation procedure involved, first, the decontamination of the fractionation system by flushing with cleaning solution at 1 mL/min flow rate. Next, the system was washed copiously with sterile, demineralized water at the same flow rate. Cells, MSCs in particular, can adhere to plastic: to block non-specific interaction sites on the plastic walls, the fractionation system was flushed at 0.5 mL/min with a sterile coating solution. Finally, it was filled with sterile mobile phase. All solutions were provided by Stem Sel Ltd.

For the study, mononuclear cells from BMC were diluted to a final concentration of 8 × 10^6^ cells per ml and 100 µL was injected. Cells were automatically re-dispersed 3 times to homogenize the suspension and eluted at a flow rate of 2 mL/min with a relaxation time of 3 min.

### 2.4. Optical Analysis

Eluted cells were monitored using a micro-camera detector, and the count-software (Stem Sel Ltd., Bologna, Italy) generated the fractogram. Dimension inclusion/exclusion criteria were set by the operator to refine the counting procedure. The dimension was set from 7 µm to unlimited µm with an average of 14 µm, to distinguish single cells from cell aggregates. The software was therefore able to recognize sizes ranging from small cells to large cell aggregates. Three captured images of eluting cells from each fraction were post-processed to measure the cell area using free imaging software Fiji (Image J software v 1.44p, NIH) with the ‘analyze-particles’ feature.

### 2.5. Analysis and Cell Collection

For every sample, cells were first analyzed to obtain a patient-specific fractogram and identify the fractions to collect. Consecutive analyses were run to increase the number of collected cells per fraction. The fractionated cells, collected in 50 mL tubes, were centrifuged and, subsequently, cell pellets were pull together; the cell number was counted using crystal violet solution (Sigma, St. Louis, MO, USA) to identify exclusively mononuclear cells and exclude RBCs. The percentage of enrichment was calculated, dividing the number of recovered cells, from each fraction, by the total number of injected cells.

Downstream analysis, CFU-F assay, differentiation assay, and visualization of physical parameters by flow cytometry were performed with freshly collected cells; to obtain the right cell number for MSCs phenotype by flow cytometry analysis, cells needed to be expanded in vitro.

Cell recovery for each fraction was compared to the integral of the output fractogram, the area under the curve. The integral was multiplied by 8 to obtain an approximation of the total number of cells because the camera framed one-eighth of the fluidic device’s area. Every run of all individuals was analyzed, summed, and compared to cell recovery.

### 2.6. Physical Characteristics

Twenty thousand cells from fresh BMC were analyzed by flow cytometry to detect physical parameters, to visualize cells cloud on FSC and SSC plot.

### 2.7. Colony-Forming Units-Fibroblast Assay (CFU-F)

The clonogenic ability of the different fraction was determined by a low-density CFU-F assay.

A total of 9500 mononuclear cells/cm^2^ collected from each fraction and cells from BMC (used as internal control, CTRL) were seeded in 9.5 cm^2^ dishes in expansion medium minimum essential medium Eagle alpha modification (α-MEM, Gibco BRL, Rockville, MD, USA) supplemented with 15% of foetal bovine serum (FBS, Euroclone S.p.A., Milan, Italy) and 1% penicillin-streptomycin (Gibco). The medium was changed twice a week. At 10 and 20 days and 14 days for the preliminary test, cells were fixed in methanol and stained with Crystal Violet (Sigma Aldrich). An aggregate containing more than 50 cells was considered as a colony originating from one cell. The number of colonies was counted using an inverted light microscope.

### 2.8. Phenotype Characterization

Cells from each fraction and CTRL were expanded for one passage and then analysed for the expression of mesenchymal (CD73, CD90, CD105) and haematopoietic (CD14, CD20, CD34, CD45) markers using the human MSC Phenotyping Kit (Miltenyi Biotec GmbH, Bergisch Gladbach, Germany). This kit was developed for the standardized identification and phenotyping of cultured human MSCs by flow-cytometry based on the defined ISCT standards. Tubes were read with FACS Canto (BD Biosciences, San Jose, CA, USA). The results were analysed with FlowJo software (FlowJo v 1.44p, LCC).

### 2.9. Differentiation Capacity

A total of 150,000 mononuclear cells from each fraction and CTRL were seeded onto a 12-well plate in expansion medium. The medium, procedure, and quantification are explained in the Supplementary Material.

Briefly, for chondrogenic differentiation, the medium was replaced after 24 h with chondrogenic medium, while for osteogenic differentiation, cells were immediately cultured in differentiation medium. Media were changed twice a week and cells were evaluated at 21 and 28 days. To assess differentiation, AlcianBlue and Alizarin Red staining were performed, respectively, for chondrogenic and osteogenic differentiation.

### 2.10. Statistical Analysis

All results were plotted using GraphPad Prism software, and a multi-parametric one-way ANOVA test was run (* *p* < 0.05, ** *p* < 0.01, *** *p* < 0.001, **** *p* < 0.0001).

## 3. Results

### 3.1. Fractionation Method Development

A method development was carried out in order to achieve an optimal cell recovery and cell enrichment of the putative MSCs in one fraction; in particular, the flow rate and relaxation parameter were studied.

**Figure 2:**
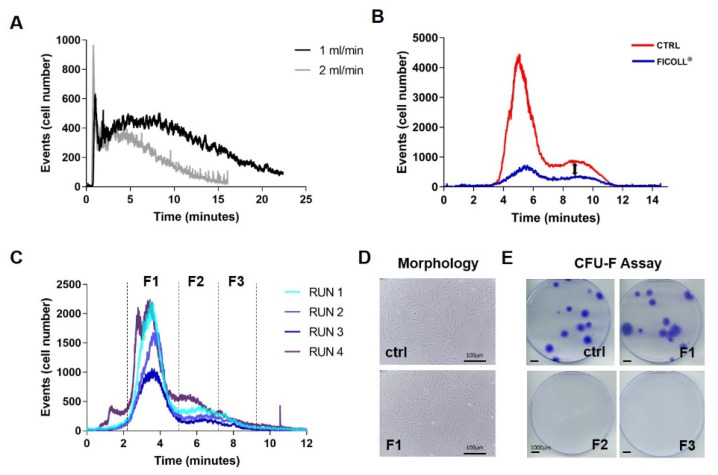
Optimization protocol for the isolation of bone marrow cells. (**A**) Two cell preparations of bone marrow cells, density gradient separation (Ficoll^®^) and IOR concentration step (CONC), were analysed at flowrate of 1 and 2 mL/min, giving an identical profile. (**B**) The two preparations were then tested adding the feature of the relaxation time of 3 min and run at a flowrate of 2 mL/min. This protocol gave a different profile, showing two main populations given by the two peaks. Bone marrow concentrated (CTRL) showed higher intensity in the first peak, which contained mainly RBCs, and also in the second one. Due to higher peak intensity not to mention avoidance of extra cell manipulation, the IOR concentration protocol was used for cell preparation. (**C**) One clinical sample was analysed to collect cells for different time interval (F1: 2–5 min; F2: 5–7 min; F3: 7–9 min) and the repetition of 4 analyses (RUN) showed reproducibility. Once cells were plated, only F1 cells attached to plate showed same morphology of CTRL (**D**) and showed ability to form CFU-F (**E**).

A suspension of 6 × 10^6^ cells per ml was prepared, and 100 µL were injected per analysis with a flowrate of 1 and 2 mL/min. Cells eluted with a positively skewed profile (Figure 2A) and doubling flow rate did not change the analysis resolution. Moreover, no difference was identified between samples obtained by Ficoll^®^ or IOR-G1 concentration step (data not shown). Conversely, adding a relaxation time of 3 min changed the profile (Figure 2B). Two main peaks were observed, and a difference in the intensity was evident between the two preparations: higher for the concentration method compared to Ficoll^®^ preparation. Cells were recovered from the two main fractions and the presence of RBCs was identified mainly in the first one, which explained the lower intensity for the Ficoll^®^ preparation because of the MNCs enrichment and consequent RBCs depletion of this procedure. The opposite, few RBCs and many cells, was observed in the second fraction with a lower intensity for the Ficoll^®^ preparation probably caused by cell loss from the several washing–centrifugation steps. Therefore, we selected the concentration method because of its higher second peak and to avoid extra cell manipulation step. An additional clinical sample processed with the IOR-G1 system was analysed, performing multiple runs to obtain a higher number of collected cells (Figure 2C). The sample was divided in 3 fractions (F1, F2, and F3), and cells were separately collected in sterile tubes for morphological and CFU-F analysis. By the overlapping profiles obtained from different injections, the system proved to maintain reproducibility among the run. When cells were plated, only F1 cells attached to the plastic surface and showed the same fibroblastic morphology of gold-standard culture. Freshly isolated cells were also plated to assess the clonogenicity, one of the gold-standard assays to define MSCs; as for the morphological aspect, only F1 cells showed CFU-F ability (Figure 2E). Therefore, this protocol was further used for our study to isolate MSCs directly from BMC.

### 3.2. Celector^®^ Bone Marrow Concentrate Profile

**Figure 3:**
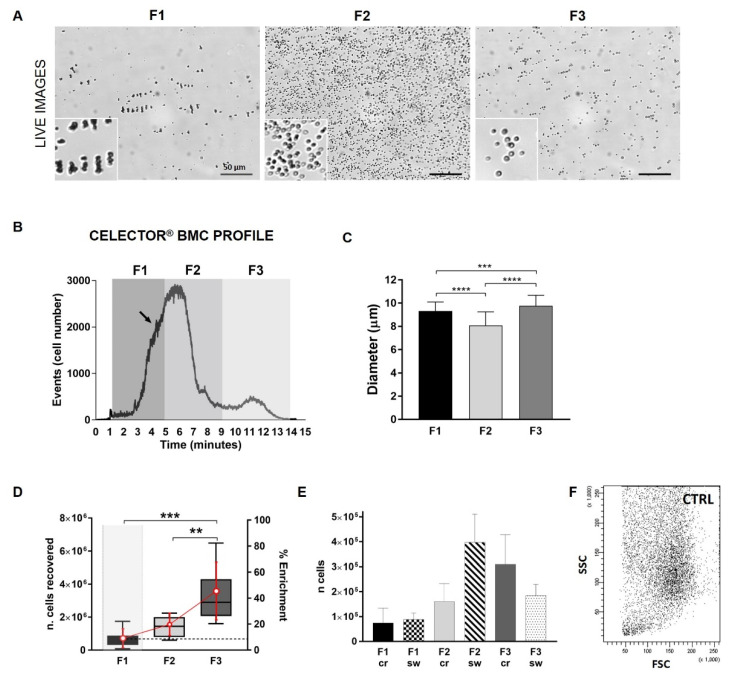
Label-free separation of fresh bone marrow cells. (**A**) Representative images from F1, F2, and F3 captured during the analysis (screenshots) (scale bar 50 µm). F1 presents many cell aggregates; in F2, RBCs are predominant, and have a more defined surface in F3 cells. (**B**) Representative Celector^®^ profile of fresh BMC. Fractogram represents the number of counted cells in correlation with the elution time in minutes. Unretained cells and debris elute at around 1 min and were discarded. Time intervals of the three collected fractions are shown in the fractogram graph (F1: 1–5 min; F2: 5–9 min; F3: 9–14 min). (**C**) Cell diameter measurement of eluting cells using ImageJ software and “analyze particles” plugin. Three screenshots per fraction were used and diameter is expressed in µm. Cell aggregates were excluded to obtain only single-cell dimension and F1 cells had a diameter of 9 µm; F2 cells, mostly RBCs, had a diameter of 8 µm; and F3 had a diameter of 9.7 µm. (**D**) Graph representation of the average cell recovery for each fraction for 8 samples analyzed and the percentage of cell enrichment of each fraction compared to the total number of cells injected into the system. Recovery and enrichment raised from F1 to F3. (**E**) Quality-control feature of counting software of Celector^®^ to identify cell recovery for a specific fraction. Cell number of each fraction was measured by the integral of the area designed by the profile (cells vs. time) (F1-2-3 sw) and compared to cell number from crystal violet count (F1-2-3 cr). Every run of each individual was calculated and then average was graphed. Cell count for F1 was very similar between the two systems while F2 cell counted by the software (F2 sw) was higher due to presence of RBCs which are not manually counted by Crystal violet staining. F3 sw count gave a lower number because of a technical limitation of miscalculation due to closing eluting cells which were considered single cells instead of a group. (**F**) Flow cytometry data of fresh BMC show great heterogeneity among cells. No distinct populations were observed if only physical parameters were used. All data are represented as mean ± SD, multi-parametric one-way ANOVA test: ** *p* < 0.01, *** *p* < 0.001, **** *p* < 0.0001.

Eight samples of BMC were harvested and resulted viable from cell counting. Cells were injected into Celector^®^, and the typical fractogram was obtained (Figure 3B). Profile comparison from all individuals confirmed reproducibility (Supplementary Figure S1). During fractionation analysis with Celector^®^, unretained cells, likely dead cells and debris, were eluted after 60 s from the start of the analysis (void). A deep look into the live images of eluting cells underlined the presence of cells, many in aggregates which eluted before the main peak of RBCs (Figure 3A). The presence of these cells was also captured by the counting system, showing a small hump just before the first peak (Figure 3B, arrow). Sorting was arranged to recover these cells and divide them from the majority of RBCs. Eluted cells were divided into three fractions based on eluting time: fraction 1 (F1) approximately from 1 to 5 min, fraction 2 (F2) from 5 to 9 min, and fraction 3 (F3) from 9 to 14 min. For each sample, 10 consecutive analyses were performed: the first was run to define the individual’s fractogram and sample collection intervals, and the following 9 identical runs to obtain a higher cell number per fraction. BM contains a variety of cell types and sizes. In the majority of the cases, F1 contained many cell aggregates, and when single cells were analysed using ImageJ software, the average diameter was around 9 µm. F2 cells had a lower dimension of 8 µm, similar to RBCs, and F3 cells appeared to have sharper contours and a diameter of 9.7 µm (Figure 3C). Moreover, this result showed how the cell density is also an important characteristic for cell separation because, based on principles, smaller cells elute later in the analysis; therefore, the cell density of F1 could be an important factor among the ultrastructural characteristics that influenced the sorting process [16]. Moreover, cells derived in F1 are indeed smaller; however, because they are mostly in an aggregate form, they reach a higher position across the channel thickness, acquiring a higher velocity and, consequently, an early exit. Right after sorting, cell recovery for each individual was similar, increasing from F1 to F3 (Figure 3D, with a percentage of enrichment of 10% for F1, 20% for F2, and 45% for F3, and a general cell recovery set at 75%. Interestingly, the higher number of recovered cells in F3 did not correspond to a higher ability to adhere to plastic surfaces, one of the definitions of MSCs. Few cells attached to the culture plate from F2 and F3 and replicated very slowly. F1 cells adhered and proliferated in every sample, confirming the preliminary results. The new sorting intervals were more efficient to obtain only adherent cells and exclude all unwanted RBCs.

In order to explore the predictivity of the fractogram, cell numbers for each fraction were extrapolated by fractogram area for each individual (Figure 3E). Number of F1 cells were similar between the two systems, cell counting and software analysis, while F2 cells were much higher in the software version because the majority of F2 cells are RBCs, which are counted by the software but are excluded by the crystal violet staining. The software counted more cells in F3 compared to F1, which is in accordance with the cell recovery trend observed, but the F3 value was underestimated compared to the cell recovery number. This could be explained by an instrumental limitation in its first version; F3 cells eluted in small groups and the image software could have counted the groups of closing cells as single cells.

### 3.3. Representation of Cells Physical Parameters

Fresh BMC cells were analysed by flow cytometry as one of the gold-standard techniques to analyze cells in their size and complexity in order to compare it with Celector^®^ data output. BMC cells were plotted using the FSC/SSC parameters, and we observed a heterogeneous population of cells that were very broad in dimension (FSC parameter) and intracellular complexity (SSC parameter) with no clear distinction of different populations (Figure 3F). It is impossible by flow-cytometry, using only physical parameters, to distinguish the multipotent MSCs.

### 3.4. Morphological and Phenotypical Analysis

**Figure 4:**
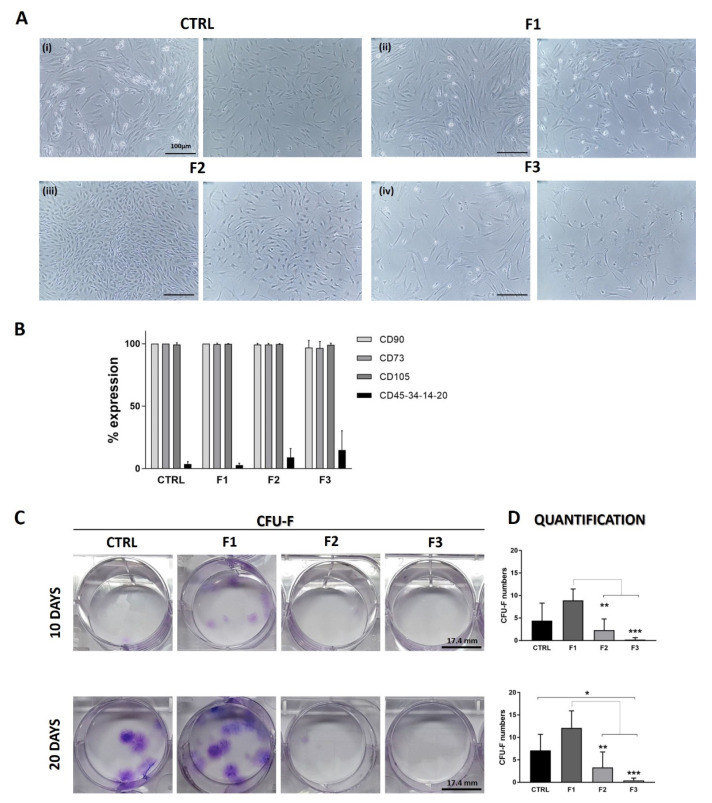
Morphology and phenotype of MSCs collected from the three fractions and their clonogenic potential. (**A**) Representative images of expanded cells from control (CTRL), F1, F2, and F3 fractions after 10 days of culture. Cells from CTRL and F1 showed the typical fibroblastic-like shape and colonies with epithelial morphology were observed in F2 cell culture, with colony growing in a spiral form and cells showing a wide cytoplasm and long extensions. Cells from F3 were very few, did not form colonies, and had a wide cytoplasm (scale bar: 100 µm). (**B**) Flow cytometry analysis showed a high and homogenous expression of mesenchymal markers CD90, CD73, and CD105 in cells from all fractions but an increase in the hematopoietic markers CD45, CD34, CD14, and CD20 in cells from F2 and F3. (**C**) Cells collected from each fraction and control were plated at low density to test capacity to form CFU-F. Assay was performed at 10 and 20 days. Colonies were stained with Crystal Violet and quantification of the number of colonies in each sample was graphed (**D**). After 10 days, F1 cells displayed higher capacity to form CFU-F compared to CTRL (8.9 vs. 4.4 CFU-F, *p*= 0.0708), and it was maintained after 20 days. Very few colonies were observed in F2 and none in F3 (mean ± SD, multi-parametric one-way ANOVA test: * *p* < 0.05, ** *p* < 0.01, *** *p* < 0.001).

Collected cells from each fraction and CTRL were expanded in culture to assess their morphology and proliferation. Morphological difference was noted among cells from different fractions. F1 cells resembled CTRL cells (Figure 4A(i)), with a typical fibroblastic-like shape and growing in colonies (Figure 4A(ii)). F2 cells still expressed fibroblastic-like shape and an arrangement of spiral-shaped growth (Figure 4A(iii)), while F3 cells looked different with a wider cytoplasm, irregular contour and long pedicles extruding from the cell body (Figure 4A(iv)). One of the main observations was the number of adherent cells per fraction: F1 contained more adherent cells and grew faster in culture, so it probably contained the most proliferative clones; few cells from F2 attached to the plate and slowly grew, whereas only a couple of cells from F3 adhered to plastic and had a slow replicative pace. F1 cells reached confluence in 20 days, like CTRL, while F2 and F3 cells reached less than 50% of confluence in approximately 40 days.

Expanded cells were detached and stained for mesenchymal and hematopoietic markers for flow cytometry analysis following ISCT standards (Figure 4B). Cells from each fraction expressed the same percentage of the mesenchymal markers (CD90, CD105, CD73: 100%), while the combination of the hematopoietic markers increased from F1 to F3 (Figure 4B), suggesting the presence of adherent cells of hematopoietic origin [17]. These results confirmed that the canonical surface protein expression of MSCs cannot be the only criterion to define MSCs.

### 3.5. Clonogenic Activity

Cells from each fraction and CTRL were tested for their clonogenic activity based on the CFU-F assay as one of the definitions for MSCs. F1 cells demonstrated clonogenic ability, and we noted a clear trend of higher clonogenic potential in F1 compared to CTRL already after 10 days, confirmed after 20. The colony-forming capacities in F2 and F3 were very different from F1. CFU-F were very low in F2 samples, and almost no colonies were present in F3 (Figure 4C). The few CFU-F colonies found in F2 were likely derived from the F1 peak’s tail.

### 3.6. Differentiation Capacity

The third requisite to define MSCs for ISCT is their ability to differentiate towards the mesenchymal lineages. We focused on chondrogenic and osteogenic differentiation ability because of the clinical perspective of this study and the use of BM-MSCs for osteochondral regeneration.

**Figure 5:**
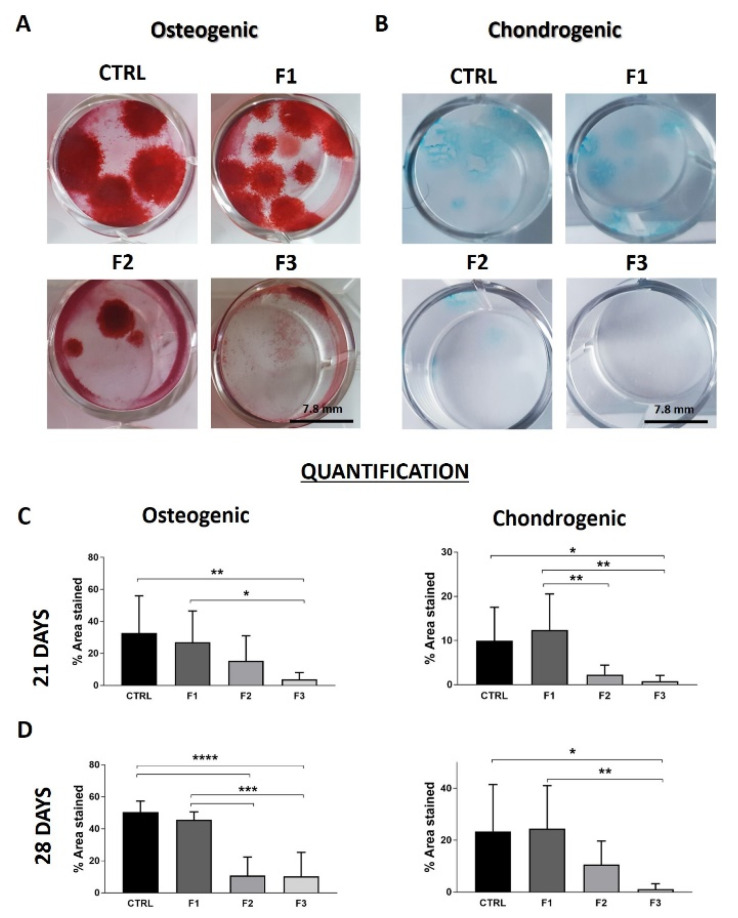
Differentiation into osteogenic and chondrogenic lineage for cells derived from F1, F2, F3, and CTRL. (**A**) Representative images of Alizarin staining for the osteogenic differentiation after 28 days of culture in differentiation medium. (**B**) Alcian blue staining for proteoglycan formation in cells derived from F1, F2, F3, and CTRL after 28 days of culture. Semi-quantitative analysis was performed measuring stained area by Image J software. Results were calculated for differentiation for osteogenic and chondrogenic differentiation after 21 days (**C**) and 28 days in culture (**D**) (mean ± SD, multi-parametric one-way ANOVA test: * *p* < 0.05; ** *p* < 0.01, *** *p* < 0.001, **** *p* < 0.0001).

We proved that only F1 cells have a differentiation potential towards osteogenic and chondrogenic lineage compared to the other two fractions. F2 cells poorly differentiated towards the two lineages, and F3 cells did not show any differentiation ability (Figure 5A,B). We observed that the stained area of F1 cells was more homogenous among individuals, with a symmetrical distribution of the stained area compared to the CTRL (skewness: chondrogenic F1 vs. CTRL: 0.7 vs. 1.5; osteogenic F1 vs. CTRL: 0.72 vs. 1.66).

## 4. Discussion

The use of MSCs in clinical trials is increasing but methods to isolate and produce cells among centres is largely heterogeneous [1]. Donor-to-donor variability [18] and different isolation/culturing protocols of primary BM-MSCs often result in the heterogeneity of clinical outcomes [19]. MSCs are defined by their surface markers expression and ability to form colonies and differentiate towards mesenchymal lineages. The International Society of Cell Therapy (ISCT) proposed CD90, CD105, and CD73 as the essential markers to define MSCs and lack of expression of CD45, CD34, CD14 or CD11b, CD79α or CD19, and HLA-DR surface molecules [6]. A pre-enrichment step using magnetic beads is necessary before multi-labelled sorting [20], even though these markers are not specific and can also be expressed by endothelial cells and fibroblasts [21,22]. Cells isolated by plastic adherence and then characterized for several mesenchymal markers and differentiation potential in vitro/in vivo cannot discriminate the best BM-MSCs population since even single strain populations differ in phenotype and multipotency [19]. To overcome these shortcomings, we established a new method to isolate MSCs from freshly harvested bone marrow concentrate (BMC), which guarantees to a greater extent to maximize the stem cell presence. The new isolation method is characterized by a label-free technology called Celector^®^. Celector^®^ can be considered a “cell-chromatograph”: cell populations are sorted according to their intrinsic physical properties (dimension, morphology, density). Different cell populations are eluted at different times and collected, obtaining a population that is homogeneous in its physical characteristics. We first optimize the isolation protocol, acting on different flowrates and stop-flow features using two cell preparations: density gradient and BMC. The use of BMC allowed a higher cell recovery as shown by peak intensity compared to density gradient step; it avoids multiple washing/centrifuge steps that could cause cell loss, and clinically, its use is increasing for osteo-cartilage regeneration and for the treatment of several orthopaedic pathologies [23].

The instrument was able to discriminate three main populations within BMC, and only one demonstrated all stemness characteristics defined by the ISCT, namely F1. F1 cells were the only ones able to adhere to plastic, form CFU-F, and differentiate towards mesenchymal lineages.

The microfluidic system demonstrated an optimal sorting resolution with 75% of cell recovery while preserving viability and proliferative and multipotent cell characteristics. The system was continuously running to obtain a high cell number, and it did not affect the sorting performance. The preservation of stem cell integrity and potential is imputed to the low mechanical and shear stress during the separation process.

Moreover, the innovative approach visualizes and sorts cell aggregates, which are usually an obstacle for sorting technologies, increasing the isolation of MSCs. These cell aggregates in F1 could be the hematons, cell aggregates isolated from BM composed of hematopoietic and MSCs [24,25,26]. The MSC enrichment in the F1 was 9% of the total BMC, a very interesting datum since it is known that MSCs from bone marrow are around 0.1%. This higher percentage could be explained by a higher efficiency in sorting proliferative and lively cells from the niche, in single or aggregate state. F1 cells showed a higher trend in their clonogenic potential compared to standard culture. We hypothesized that the exclusion of the other 65% of recovered cells from F2 and F3 could positively affect this result. F1 cells also showed a more homogeneous differentiation ability towards osteogenic and chondrogenic lineages among individuals compared to CTRL.

Notwithstanding only a few cells from F2 and F3 attached to plastic culture dishes and had a low proliferative rate, they were the majority of recovered cells after the isolation procedure, 20 and 45%, respectively. These cells had a heterogeneous morphology, with the arrangement of spiral-shaped growth cells and cells with wider cytoplasm [19]. Previous works from the Prockop group also showed differences in morphology among BM-MSCs [27,28,29]: smaller cells were isolated by physical parameters with flow cytometry but did not express a different percentage of MSC markers compared to the whole sample as confirmation that no surface epitopes are able to distinguish subpopulations in different preparations of MSCs. We confirmed this observation: adherent cells from F2 and F3 equally expressed mesenchymal markers compared to F1 and CTRL. However, F2 and F3 cells showed a higher percentage of hematopoietic markers, 9 and 15%, and it is known that the hematopoietic fraction of BM-MSCs is around 20–30% in human BM-MSCs cultures [17], which is exactly the sum of expression of F2 and F3 cells. These fractions are likely to contain the majority of this sub-population.

Celector^®^ shows an added value in the isolation of BM-MSCs; it cleans the red blood cells (RBCs) from the BM. The majority of RBCs were collected in F2, so BM-MSCs culture was depleted from these unwanted cells. RBCs can obstruct cell adhesion, and it was proven that they can affect the functionality of isolated BMCs and impaired organ recovery in patients with acute myocardial infarction [30].

One of the data outputs of Celector^®^ is the sample fractogram and live images, a fingerprint analysis of the cell composition that discriminates differences in cell populations and gives immediate feedback on the presence of MSCs. The micro-camera detects, in live mode, the variety of cell population, discriminating between single cells or aggregates and unwanted unretained material. Live images and post-processing analysis are interesting features that need to be upgraded, but they already offer extra information on the cell morphology. In addition, the cell number extrapolated from fractogram from F1 was comparable to its cell recovery. Therefore, the presence of the hump in the first part of the profile is a sign of the presence of MSCs in that specific patient and could be used as a predictive outcome for cell usage.

In conclusion, we tested and proved the efficacy of a new technique for the isolation and enrichment of BM-MSCs from raw samples. The process is reproducible among patients, with a characteristic profile. Cells derived from all three fractions highly expressed mesenchymal markers, but the technology could discriminate the fraction containing the actual multipotent MSCs based only on their native physical properties. Moreover, the system allowed purifying raw samples, with a depletion of differentiated, hematopoietic cells, and RBCs. Further studies need to be performed in order to obtain insights into the stemness and paracrine potential of the selected cell population. These are very promising results which open interesting perspectives for the use of this technology to improve stem cell isolation and quality control from raw clinical samples. The technology could be implemented in order to process a higher number of cells to obtain the sufficient cell amounts for preclinical applications within less cycles and to ameliorate cell recognition and cell counting to be used when there is a necessity to compare the quality of MSCs due to differences in harvesting techniques, isolation, culture conditions, and different harvesting sites, resulting in different MSC yields [31,32].

## 5. Patents

Celector^®^ is based on a technology patented in Italy (No. IT1371772, “Method and Device to separate totipotent stem cells”), in the USA, and in Canada (No. 8,263,359 US en. CA2649234, “Method and device to separate stem cells”). Stem Sel^®^ also has an Italian patent (IT1426514, “Device for the Fractionation of Objects and Fractionation Method, allowed 2016).

## Data Availability

Not applicable.

## Supplementary Materials

The following supporting information can be downloaded at: https://www.mdpi.com/article/10.3390/bioengineering9020049/s1: Figure S1: Celector^®^ profiles of fresh BMC from the eight patients.
