# Supplementary figures and images for: Effective Label-Free Sorting of Multipotent Mesenchymal Stem Cells from Clinical Bone Marrow Samples
Source: Bioengineering (Basel). 2022 Jan 22;9(2):49. doi: 10.3390/bioengineering9020049 (PMC8869157; doi:10.3390/bioengineering9020049)

## Supplementary Materials

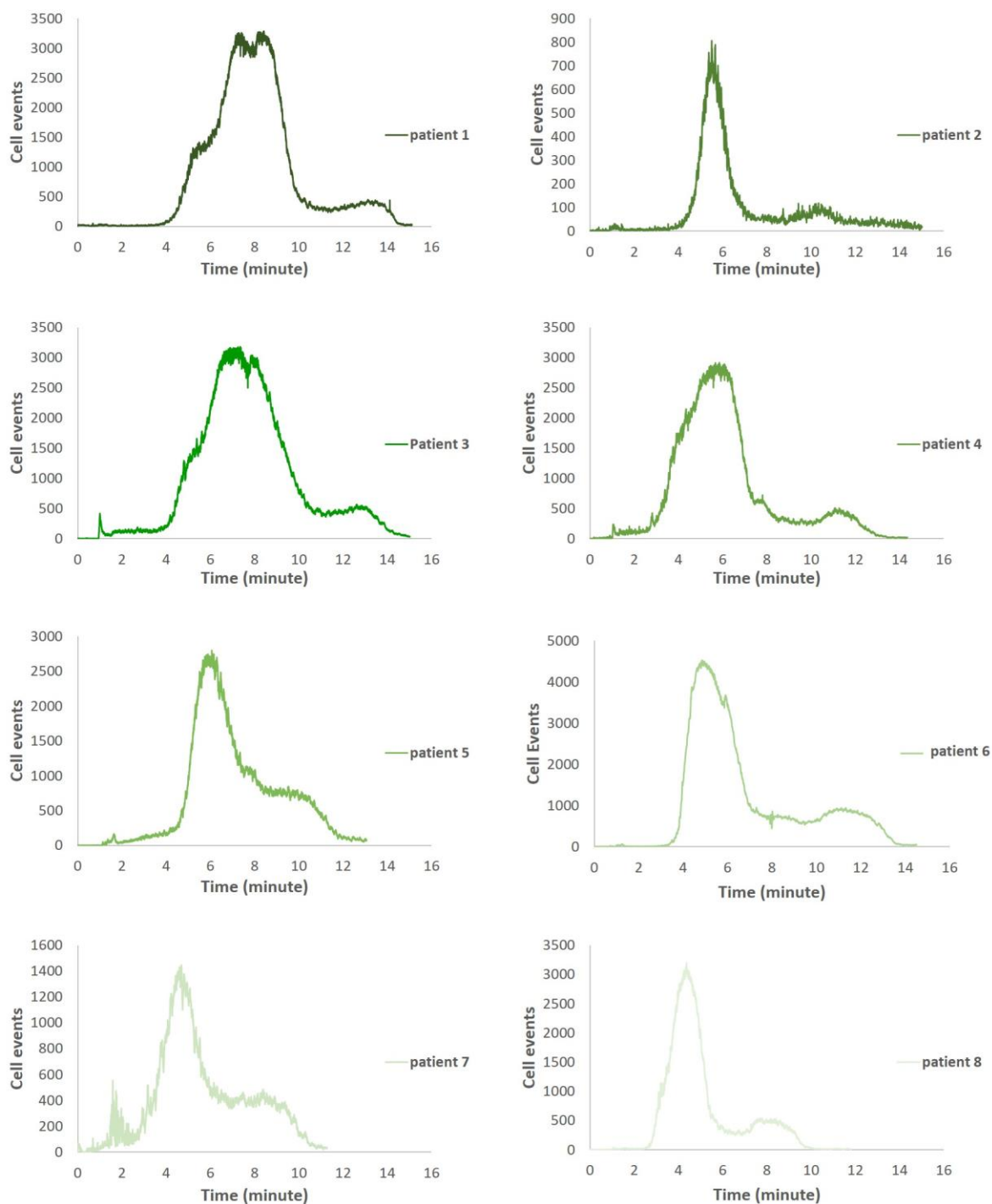

Figure S1. Selector® profiles of fresh BMC from the eight patients.

Supplement: Supplementary file 1 [file bioengineering-09-00049-s001.zip › bioengineering-1555610-supplementary.pdf]
